# Editorial: Emerging talents in clinical diabetes

**DOI:** 10.3389/fendo.2023.1284900

**Published:** 2023-09-11

**Authors:** Paulo Matafome, Ping Wang

**Affiliations:** ^1^ Polytechnic University of Coimbra, Coimbra Health School, Coimbra, Portugal; ^2^ Coimbra Institute for Clinical and Biomedical Research (iCBR), Center for Innovative Biomedicine and Biotechnology (CIBB), Faculty of Medicine, University of Coimbra, Coimbra, Portugal; ^3^ Clinical Academic Center of Coimbra (CACC), Coimbra, Portugal; ^4^ Precision Health Program, Michigan State University, East Lansing, MI, United States; ^5^ Department of Radiology, College of Human Medicine, Michigan State University, East Lansing, MI, United States

**Keywords:** diabetes, emerging talent, diagnosis, prognosis, artificial intelligence

Globally, students are engaging in significant diabetes research as an integral part of their education. Regrettably, much of this valuable work remains hidden from the broader public due to students’ apprehensions about peer review. At Frontiers, we view peer review as a cooperative endeavor. Our interactive peer-review system is meticulously designed to offer practical assistance and positive input to researchers. Our dedicated Topic Editors are passionate about nurturing emerging talents and encouraging student researchers to achieve publication success.

The research showcased in this work underscores the caliber and variety of student researchers in the realm of diabetes. This Research Topic aimed to publish the studies conducted by student researchers encompassing areas such as: 1). Explorations in the clinical science of diabetes, encompassing the etiology and pathogenesis of major diabetes subtypes. 2). Examination of associated complications of diabetes. 3). Investigation into dysfunctions affecting the major organs contributions to dysmetabolism.

In this Research Topic, researchers had the opportunity to publish their innovative research related to these abovementioned topics. Gu et al. described the differences in spexin levels between newly diagnosed patients with diabetes according to their body mass index (BMI). This 14-amino acid protein has been associated with improved metabolic function and its levels were observed to be significantly decreased in patients with higher BMI (1). Interestingly, the study by Gu et al. demonstrated that serum spexin levels increased upon body weight loss, which indicated that when the metabolic disorder of type 2 diabetes mellitus (T2DM) patients improved, the improvement of inflammatory factors would also affect the level of serum spexin. However, the mechanisms underlying the associations of spexin with obesity and diabetes remain to be further determined. The study by Chen et al. aimed to understand the usefulness of hemoglobin A1c (HbA1c) for diabetes diagnosis in patients with pancreatic diseases. The study demonstrates that in these patients the current cut-off HbA1c values for diabetes diagnosis may not be sufficient. The authors suggest that 6.0% may be a better value for diabetes diagnosis for future clinical use in these patients.

Lately, there has been a growing inclination toward employing artificial intelligence algorithms, encompassing machine learning and deep learning, for prognosticating disease progression and outcomes - diabetes being no exception (2). In this Research Topic, several authors published their finding regarding automated methods for different problems related to diabetes. Liu et al. developed a machine learning-augmented algorithm to predict diabetes in community and primary care screenings. On the other hand, Yun et al. developed an automated method to predict one-year risk of severe hypoglycemia in patients with type 2 diabetes. Importantly, machine learning was also used by Li et al. to predict the risk of medication nonadherence in patients with type 2 diabetes, aiming to improve diabetes management. Regarding diabetes complications, Wang et al. analyzed the used algorithms to predict retinal lesions in patients with diabetes through a systematic bibliographic analysis. The authors revised the algorithms able to recognize eye fundus lesions and concluded that more engineering development and involvement of the medical community are necessary for algorithm training. The lack of accuracy and efficiency of the methods currently available may also derive from the different variables present in the analysis like gender, ethnicity, stage of disease, etc. These variables are true, not only for algorithms predicting diabetes complications, but also for those predicting hyperglycemia itself. The majority of algorithms were formulated within distinct populations, posing a challenge to their effective translation into real-life contexts within other demographic groups.

In essence, this Research Topic provided a platform for emerging researchers to publish their diverse work on clinical diabetes. It’s imperative that we persist in adopting similar approaches to foster groundbreaking research by young scholars, ultimately enhancing the quality of life for diabetic patients.

## Author contributions

PM: Writing – original draft, Writing – review & editing. PW: Writing – original draft, Writing – review & editing.
